# Reference data for left ventricular filling and atrial function in children using cardiovascular magnetic resonance

**DOI:** 10.1186/s12968-023-00936-x

**Published:** 2023-06-12

**Authors:** Christopher C. Henderson, Kristen George-Durrett, Sandra Kikano, James C. Slaughter, Joshua D. Chew, David Parra, Jeffrey Weiner, Jonathan Soslow

**Affiliations:** 1 Department of Pediatrics, Vanderbilt University Medical Center, Nashville, TN USA; 2 Division of Pediatric Cardiology, Department of Pediatrics, Vanderbilt University Medical Center, Nashville, TN USA; 3 Department of Biostatistics, Vanderbilt University School of Medicine, Nashville, TN USA

**Keywords:** Diastolic dysfunction, Cardiovascular magnetic resonance, Reference values, Children

## Abstract

**Background:**

Diastolic dysfunction is associated with morbidity and mortality in multiple pediatric disease processes. Cardiovascular magnetic resonance (CMR) provides a non-invasive method of studying left ventricular (LV) diastolic dysfunction through the assessment of LV filling curves and left atrial (LA) volume and function. However, there are no normative data for LV filling curves and the standard method is time-intensive. This study aims to compare an alternate, more rapid method of obtaining LV filling curves to standard methodology and report normative CMR diastolic function data for LV filling curves and LA volumes and function.

**Methods:**

Ninety-six healthy pediatric subjects (14.3 ± 3.4 years) with normal CMR defined by normal biventricular size and systolic function without late gadolinium enhancement were included. LV filling curves were generated by removing basal slices without myocardium present throughout the cardiac cycle and apical slices with poor endocardial delineation (compressed method), then re-generated including every phase of myocardium from apex to base (standard method). Indices of diastolic function included peak filling rate and time to peak filling. Systolic metrics included peak ejection rate and time to peak ejection. Both peak ejection and peak filling rates were indexed to end-diastolic volume. LA maximum, minimum and pre-contraction volumes were calculated using a biplane method. Inter-and intra-observer variability were assessed with intraclass correlation coefficient. Multivariable linear regression was used to assess the effects of body surface area (BSA), gender and age on metrics of diastolic function.

**Results:**

BSA had the largest effect on LV filling curves. Normal LV filling data are reported for both compressed and standard methods. The time to perform the compressed method was significantly shorter than the standard method (median 6.1 min vs. 12.5 min, p < 0.001). Both methods had strong to moderate correlation for all metrics. Intra-observer reproducibility was moderate to high for all LV filling and LA metrics except for time to peak ejection and peak filling.

**Conclusions:**

We report reference values for LV filling metrics and LA volumes. The compressed method is more rapid and produces similar results to standard methodology, which may facilitate the use of LV filling in clinical CMR reporting.

**Supplementary Information:**

The online version contains supplementary material available at 10.1186/s12968-023-00936-x.

## Background

Left ventricular (LV) diastolic dysfunction is an important predictor of morbidity and mortality in adult and pediatric heart disease. It is characterized by impaired ventricular relaxation and is associated with increased risk for adverse cardiac outcomes including arrhythmias, myocardial hypertrophy, ventricular fibrosis and ischemia, progression to systolic dysfunction, and sudden cardiac death [1–4]. Diastolic dysfunction associates with morbidity and mortality in multiple disease processes, including dilated cardiomyopathy, hypertrophic cardiomyopathy, and after Fontan palliation [2, 3, 5, 6]. Cardiac catheterization is the gold standard for assessment of diastolic dysfunction, but it is invasive and expensive. It also typically requires sedation and positive pressure ventilation, both of which may affect measures of diastolic function. Non-invasive methods of characterizing diastolic function in children could improve clinical decision making and allow a better understanding of the implications of diastolic dysfunction in pediatric heart disease.

Echocardiography is commonly used to assess diastolic dysfunction in children, as it is non-invasive, readily available, and a relatively rapid means of doing so. Although there are multiple parameters to evaluate diastolic function by echocardiography, they all have limitations, which makes accurate assessment challenging [7]. Cardiovascular magnetic resonance (CMR) provides an alternate, non-invasive method of studying diastolic function via the measurement of leftr atrial (LA) volumes and function as well as LV filling curves. Data from our group suggest CMR diastology correlates with pulmonary capillary wedge pressure (PCWP) in pediatric heart transplant patients [8]. In addition, our group has demonstrated an association between CMR measures of diastolic function and arrhythmia in tetralogy of Fallot patients [9]. Although CMR assessment of diastolic dysfunction in pediatrics has significant potential, there are only limited reports of normative data in children. In addition, calculation of filling curves can be time-consuming. Thus, we propose an alternate and potentially more rapid method of generating LV filling curves that removes apical slices with poor endocardial delineation and basal slices without myocardium present throughout the cardiac cycle. The objectives of this study were to: 1) compare our laboratory’s more rapid method of obtaining LV filling curves to standard methodology, and 2) report normative pediatric CMR diastolic function data for both LV filling curves and for LA volumes and function.

## Methods

### Subject selection

This study was approved by the Vanderbilt University Medical Center Institutional Review Board. We performed a retrospective review of patients that had a normal CMR as defined by normal biventricular sizes and systolic function without evidence of late gadolinium enhancement. Of these patients, those 21 years old or younger referred for CMR for the following reasons were included: chest pain, exercise intolerance, syncope/pre-syncope, palpitations, abnormal electrocardiogram (ECG), abnormal echocardiogram, and family history of sudden cardiac death or cardiomyopathy. Patients were excluded if they had history of prior cardiac surgery, genetic syndromes, myocarditis or elevated troponin, hemodynamically significant structural heart disease (Qp:Qs > 1.5), exposure to cardiotoxic medications, prior coronavirus disease 2019 (COVID-19) infection, arrhythmia, or other chronic medical conditions. All patients retrospectively enrolled were cleared by their cardiologist. All patients with family history of cardiomyopathy were phenotypically negative, asymptomatic, had normal CMR, and had no abnormalities detected on subsequent evaluations. An additional subset of patients was enrolled prospectively as healthy volunteers to determine normal parametric mapping values. Written, informed consent was obtained for all healthy volunteers. Height and weight were measured prior to the CMR. Heart rates were taken from the heart rate calculated during the short axis stack. Blood pressure was taken prior to CMR for all clinically indicated scans but not in healthy volunteers. An initial pool of 783 patients was evaluated; a total of 96 subjects met inclusion/exclusion criteria. CMR images were obtained between 2013 and 2021.

### CMR imaging

Images were obtained on a 1.5T magnet (Avanto or Avanto Fit; Siemens Healthcare, Erlangen, Germany; Intera, Philips Healthcare, Best, The Netherlands). Balanced steady-state free precession (bSSFP) imaging was used to obtain retrospectively gated cine images in the apical two chamber, LV outflow tract, apical four chamber, and short axis views. Short axis images were obtained in a stack covering the entire LV from base to apex. Images were 8 mm thick with no gap. Typical scanning parameters were: TR = 36.5 ms, TE = 1.2 ms, flip angle 80°, voxel size 1.5 × 1.5 × 8 mm, 25 phases per cardiac cycle. Breath held images without sedation were obtained in the majority of cases, as is standard protocol in our institution. In our cohort, two patients were performed under anesthesia and two were unable to breath-hold, requiring free breathing with multiple signal averages.

### Left atrial volume and function

LA volume and function analysis was performed as previously described using 4- and 2- chamber cine images by an image analyst (KGD) using QMass (MedisSuite 2.1, Medis Medical Imaging, Leiden, The Netherlands) [10, 11]. Endocardial contours of the LA area and the LA length from mitral valve annulus to posterior LA wall were measured at maximum volume (LA_max_), minimum volume (LA_min_), and at pre-atrial contraction (LA_bac_). The time of LA_max _was defined as the last image immediately before mitral valve opening. The time of LA_min_ was defined as the first image after the closure of the mitral valve. LA_bac_ was determined by visual inspection as the last image before atrial contraction; if this could not be determined based on visual inspection, the LA_bac_ was not measured (N = 7). LA areas excluded the atrial appendage and the pulmonary veins. LA volumes were calculated using the area-length method: volume = (0.848 × area_4ch_ × area_2ch_)/([length_2ch_ + length_4ch_]/2) [12].

The LA function was determined by calculating the total LA ejection fraction (LAEF) and the LAEF for passive (LAEF_Passive_) and active (LAEF_Active_) phases. LAEF_Passive_ is defined as the difference between LA_max_ and LA_bac_. LAEF_Active_ is the difference in LA_bac_ and LA_min_. LAEF was calculated as (LA_max_-LA_min_)/ LA_max_ × 100; LAEF_Passive_ as (LA_max_-LA_bac_)/LA_max_ × 100; and LAEF_Active_ as (LA_bac_-LA_min_)/LA_bac_ × 100 [10].

### Left ventricular filling curves

Filling curves were generated by an image analyst (KGD) by contouring each phase of the cardiac cycle. Initially, the Medis artificial intelligence (AI) algorithm was utilized to contour all phases of all images. These images were then manually adjusted using our laboratory’s standard filling curve protocol, which involves removal of basal slices without myocardium present throughout the cardiac cycle and removal of apical slices with poor endocardial delineation (referred to subsequently as “compressed method”).

**Fig. 1:**
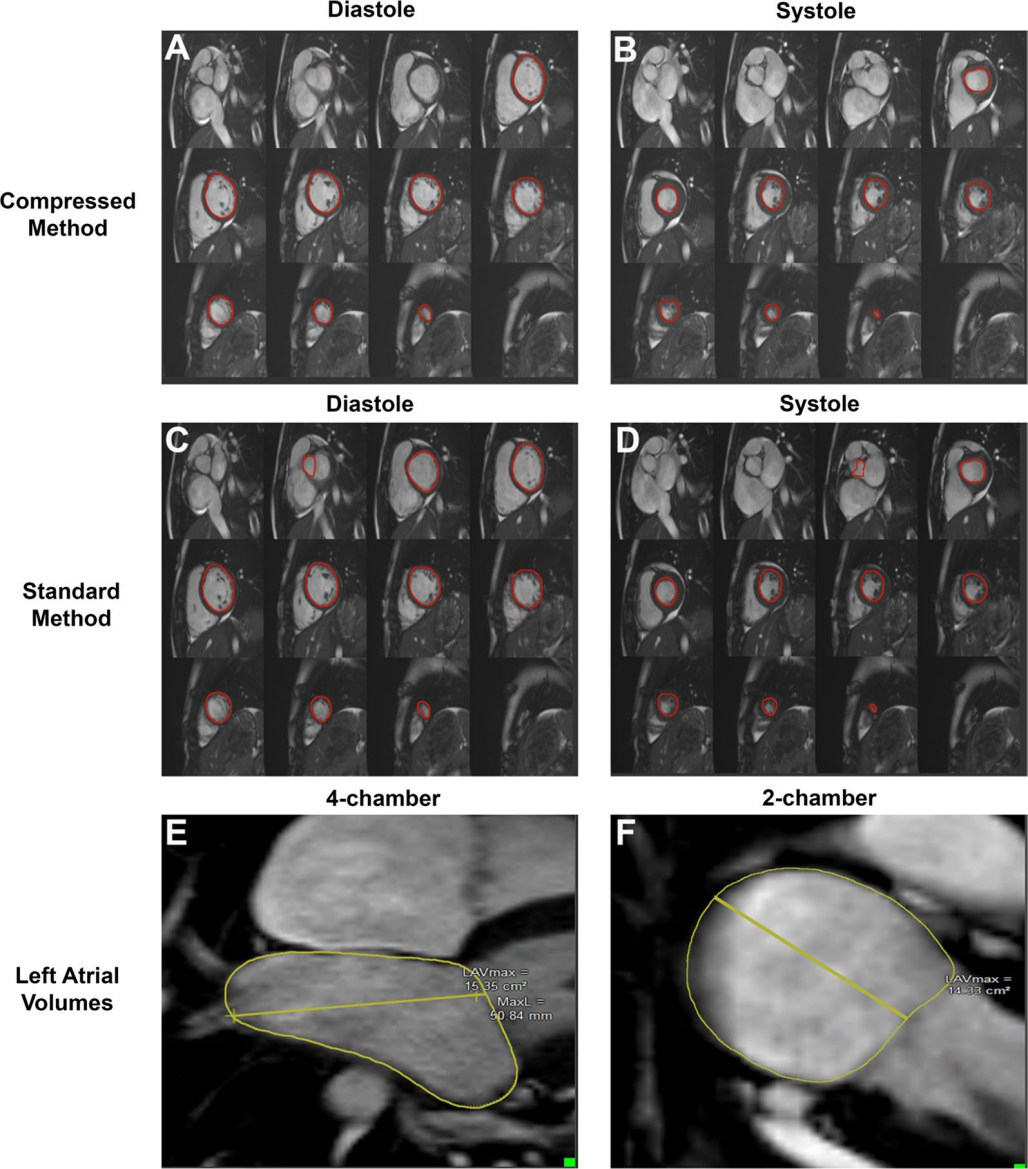
Sample endocardial contouring for compressed **A**, **B** versus standard method **C**, **D** and left atrial (LA) volume measurements **E**, **F**

Images were then re-contoured with the Medis AI algorithm such that every phase with myocardium, from apex to base, was contoured to generate filling curves as previously described (referred to subsequently as “standard method”) (Fig. 1) [13, 14]. A subset of images was reviewed by a cardiologist with over 10 years of CMR reading experience.

**Fig. 2:**
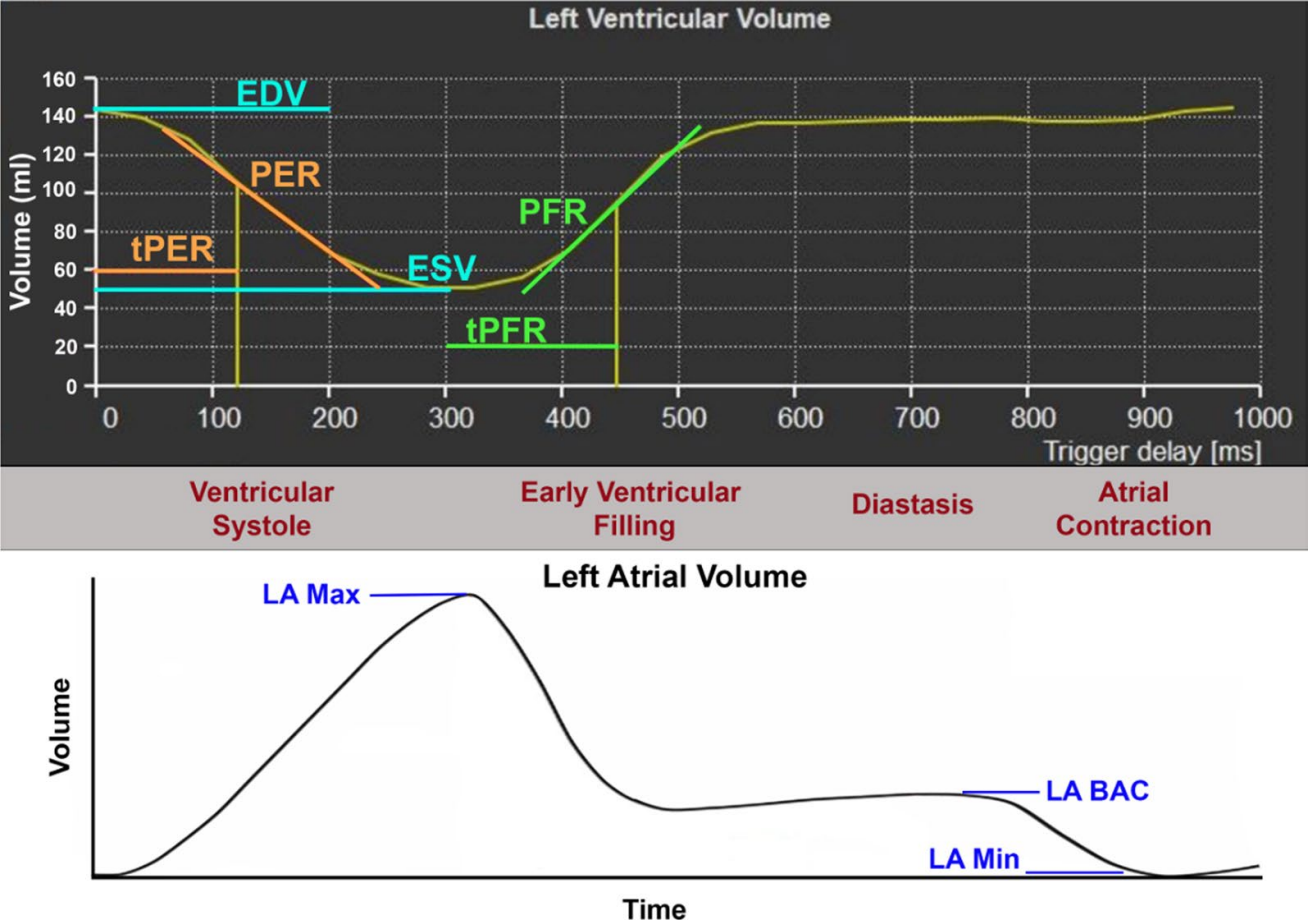
Sample LV filling curve with key indices of diastolic function. Estimated atrial filling curve is included below the sample left ventricular (LV) filling curve for reference. *BAC* before atrial contraction, *EDV* end-diastolic volume, *ESV* end systolic volume, *PER* peak ejection rate, *PFR* peak filling rate, *tPER* time to peak ejection rate, *tPFR* time to peak filling rate

For both methods, indices of diastolic function, including peak filling rate (PFR), time to peak filling (tPFR), and PFR indexed to end-diastolic volume (EDV; PFR/EDV), and indices of systolic function, including peak ejection rate (PER), time to peak ejection (tPER), and PER indexed to EDV (PER/EDV), were automatically generated by QMass. After generating a LV time-volume curve with instantaneous filling rates plotted over time, LV filling indices were defined as the following (Fig. 2):

1. PFR: maximal increase in LV volume over time, which correlates to the maximal positive slope in the volume curve occurring in early diastole.
2. tPFR: time interval from the end-systole phase to PFR.
3. PER: maximal decrease in LV volume over time, which correlates to the maximal negative slope in the volume curve occurring in systole.
4. tPER: time interval between end-diastolic phase to PER.

### Intra- and Inter-observer variability

To evaluate intra-observer variability, the same reader re-measured LA volumes and LV filling (both compressed and standard) in a subset of 25 subjects. These same 25 subjects were re-contoured by a second reader (JHS) to obtain inter-observer variability. Time to complete LV filling measurement was measured by the second reader to evaluate the length of time it would take to complete the analysis using both the compressed and standard methods.

### Statistical analysis

Demographic variables were compared using either a Wilcoxon rank-sum (continuous variables) or a Chi-square or Fisher’s exact test (categorical variables). Inter- and intra-observer variability were assessed using an intraclass correlation coefficient. The following values were used as cutoffs for weak, moderate, and strong correlation respectively: 0.20–0.39, 0.40–0.59, and ≥ 0.60 [15]. Bland–Altman graphs were used as a secondary assessment of reproducibility. The effect of age, gender, and body surface area on metrics of diastolic function was evaluated using multivariable linear regression. In order to understand other factors that might affect diastolic function, the correlation of heart rate, body mass index, and systolic blood pressure with diastolic measures was evaluated using a Spearman correlation. A non-linear model using regression splines or smooth functions of covariates was also considered, but there was no evidence that such a model would provide a better fit for the data. As such, normative data are expressed in a locally weighted scatterplot smooth curve fit to allow for some curvature, but results are reported from a simple linear model, as this was found to have adequate fit. Results are then further stratified by metrics that were significant.

Analyses were performed with STATA (version 15.1, Stata Corporation, College Station, Texas, USA). Study data were collected and managed using REDCap (Research Electronic Data Capture) electronic data capture tools hosted at Vanderbilt.

## Results

Of the 96 healthy subjects enrolled, 53 were male. Mean age of all participants was 14.3 ± 3.4 yr (range 7–21). The majority of participants self-identified as White (Table 1). There were 33 subjects who were recruited as healthy volunteers that underwent CMR for study purposes only. The remaining 63 subjects were patients referred for evaluation by a cardiologist and subsequently had normal CMR. The most common reason for cardiologist referral was chest pain or exercise intolerance, and the most frequent indication for CMR was an abnormal echocardiogram. The most common findings on echocardiogram, in conjunction with the patient’s chief complaint, that precipitated CMR were hemodynamically insignificant atrial septal defect (n = 11), concern for right sided chamber enlargement or LV non-noncompaction (each n = 3), and concern for anomalous pulmonary veins, septal hypertrophy, dilated aortic root, or mildly depressed systolic function (all n = 2). Key demographic data, reasons for referral and indication for CMR are summarized in Tables 1 and 2.

**Table 1:** Summary of demographic data for all subjects

Demographic	n (% of total)
Age range	
7–11 years	24 (25)
12–16 years	47 (49)
17–21 years	25 (26)
Gender	
Male	53 (55)
Female	43 (45)
Race	
White	63 (65)
Black	10 (10)
Other	3 (3)
Not specified	20 (21)
Body surface area (m^2^)	
0.75–1.59	34 (35)
1.60–1.89	24 (25)
≥ 1.90	38 (40)

**Table 2:** Referral reasons and cardiovascular magnetic resonance (CMR) indications for all subjects

	Reason for cardiologist referral (% of sample)	Reason for CMR evaluation (% of sample)
Chest pain or exercise intolerance	19 (20)	3 (3)
Syncope/pre-syncope	12 (13)	7 (7)
Family history of cardiomyopathy/sudden death	10 (10)	8 (8)
Atrial septal defect	5 (5)	–
Murmur	4 (4)	–
Abnormal echocardiogram	5 (5)	33 (34)
Abnormal electrocardiogram	2 (2)	9 (9)
Palpitations	3 (3)	–
Other	3 (3)	3 (3)
Recruited as healthy controls	33 (34)	33 (34)

### Reproducibility

The compressed and standard methods for assessment of diastolic filling correlated strongly for PER, PER/EDV, PFR, and PFR/EDV (ICC = 0.87, 0.82, 0.86, and 0.85 respectively, p < 0.001 for all). There was moderate correlation for tPER and tPFR (ICC = 0.50 and 0.40, p < 0.001 for both). ICC data are summarized in Table 3. Bland Altman analysis suggests an expected bias for PFR and tPFR that resolved with EDV correction (Additional file 1: Fig. S1). The intra-observer reproducibility was high for both the compressed and standard methods for PER, PER/EDV, PFR, and PFR/EDV (Table 4).

**Table 3:** Intraclass correlation coefficients (ICC) demonstrating strength of correlation between compressed and standard methods

	ICC	p-value
PFR	0.86	< 0.001
tPFR	0.40	< 0.001
PFR/EDV	0.85	< 0.001
PER	0.87	< 0.001
tPER	0.50	< 0.001
PER/EDV	0.82	< 0.001

**Table 4:** Reproducibility data for LV filling metrics by standard (a) and compressed (b) methods

	Intra-observer variability		Inter-observer variability	
	Correlation coefficient (r)	p-value	Correlation coefficient (r)	p-value
Standard method (3a)				
PER	0.93	< 0.001	0.81	< 0.001
tPER	0.59	0.002	0.57	0.003
PER/EDV	0.83	< 0.001	0.45	0.021
PFR	0.93	< 0.001	0.83	< 0.001
tPFR	0.41	0.028	-0.02	0.54
PFR/EDV	0.91	< 0.001	0.69	< 0.001
Compressed method (3b)				
PER	0.84	< 0.001	0.81	< 0.001
tPER	0.20	0.129	0.38	0.028
PER/EDV	0.85	< 0.001	0.76	< 0.001
PFR	0.85	< 0.001	0.79	< 0.001
tPFR	0.51	0.004	0.09	0.325
PFR/EDV	0.89	< 0.001	0.87	< 0.001

Inter-observer reproducibility was high for PER and PFR regardless of which method was used, but PER/EDV and PFR/EDV reproducibility were only high in the compressed method (Table 4). The reproducibility of tPER and tPFR (both intra- and inter-) was average to poor no matter which method was used. Bland–Altman graphs suggests good reproducibility with a mild bias based on the reader for both compressed and standard methods (Additional file 1: Figs. S2 and S3). Intra-observer reproducibility was high for LA_max_, LA_min_, and LA_bac_. Inter-observer reproducibility was high for LA_max_ and moderate for LA_min_ and LA_bac_ (Table 5).

**Table 5:** Reproducibility data for LA volumes

	Intra-observer Variability		Inter-observer Variability	
	Correlation coefficient (r)	p-value	Correlation coefficient (r)	p-value
LA_max_	0.80	< 0.001	0.78	< 0.001
LA_min_	0.79	< 0.001	0.66	< 0.001
LA_bac_	0.83	< 0.001	0.63	< 0.001

The time to perform the compressed method was significantly shorter than the standard method, with a median time of 6.1 IQR (4.5, 7.5) vs 12.5 min IQR (11.3, 16.1), p < 0.001.

### Normative data and multivariable regression

**Fig. 3:**
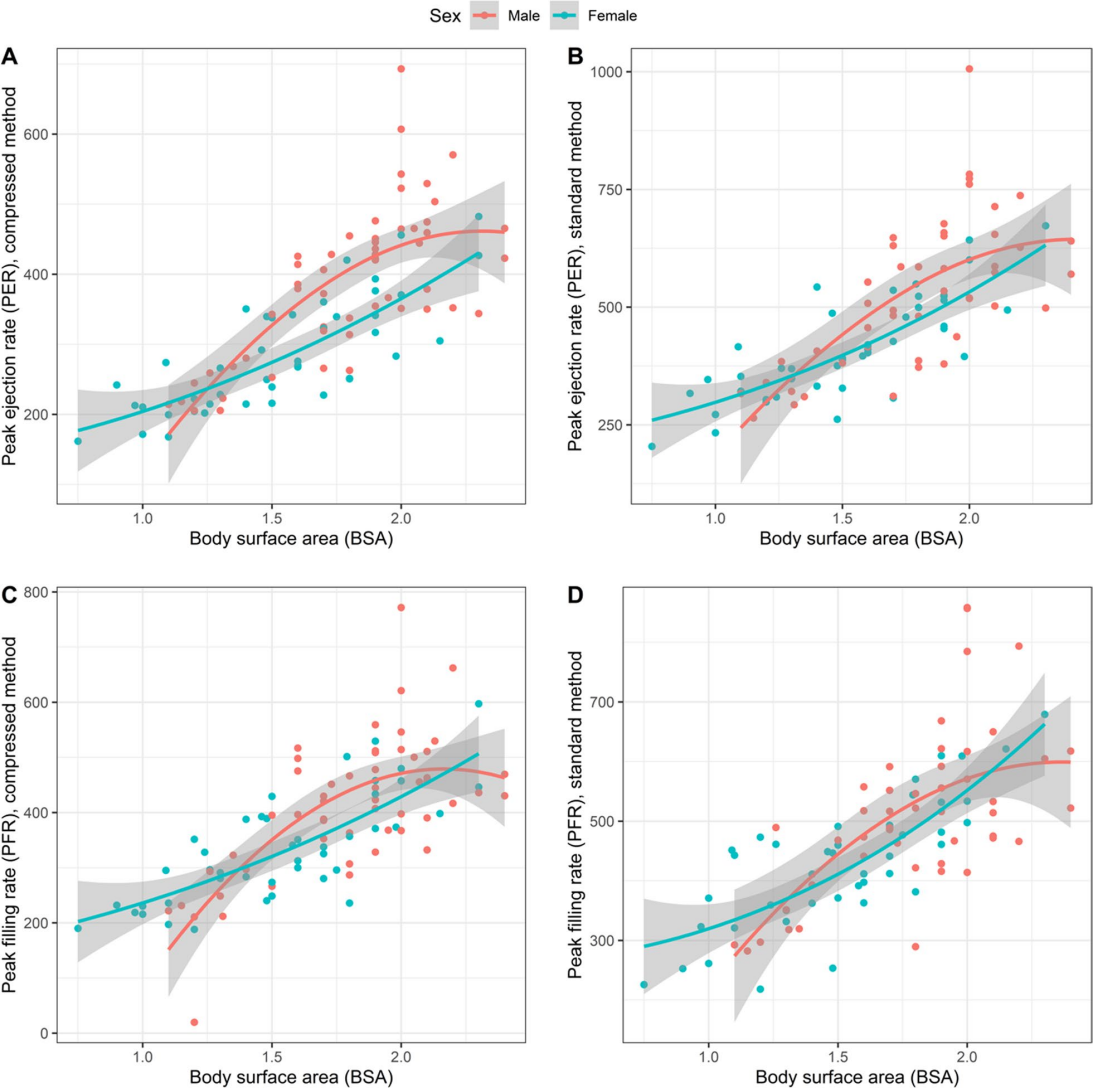
Effect of body surface area (BSA) and gender on peak filling rate (PFR) and peak ejection rates (PER). PFR and PER vary based on gender and BSA. Plots demonstrated normative data by BSA and gender in both compressed and standard methods for PER **A**, **B** and PFR (**C**, **D**)

Multivariable linear regression analysis was used to determine the effects of body surface area (BSA), gender, and age on measures of diastolic function. These results were used to determine how the normative data would be presented for each variable. Of note, age did not have an effect on filling metrics when BSA was included in the models, therefore no normative data are stratified by age. Both BSA and gender had significant effects on PER and PFR; normal values for PER and PFR are therefore categorized by BSA and gender (Table 6). The effect of BSA and gender on PFR and PER are further highlighted in Fig. 3. The equations for the multivariable regression for PER and PFR using the compressed method are:

**Table 6:** Reference values for PFR and PER categorized by BSA and gender. IQR: interquartile range

	Body surface area 0.75–1.59 m^2^ (n = 34)		Body surface area 1.60–1.89 m^2^ (n = 24)		Body surface area ≥ 1.9 m^2^ (n = 38)	
	Male (n = 11)	Female (n = 23)	Male (n = 14)	Female (n = 10)	Male (n = 28)	Female (n = 10)
Standard method						
Peak filling rate (ml/s)						
Median (IQR)	319 (293,393)	371 (321,449)	502 (458,548)	427 (394,506)	563 (483,643)	533 (479,613)
Mean ± SD	345 ± 73	369 ± 83	491 ± 72	449 ± 66	584 ± 122	550 ± 71
Peak ejection rate (ml/s)						
Median (IQR)	321 (293,381)	346 (303,387)	488 (408,586)	453 (409,526)	623 (528,672)	519 (458,646)
Mean ± SD	325 ± 51	346 ± 74	493 ± 97	455 ± 72	622 ± 122	541 ± 91
Compressed method						
Peak filling rate (ml/s)						
Median (IQR)	249 (212,297)	281 (230,341)	409 (361,469)	319 (292,353)	460 (410,514)	452 (392,492)
Mean ± SD	262 ± 59	284 ± 69	410 ± 66	330 ± 67	475 ± 96	455 ± 66
Peak ejection rate (ml/s)						
Median (IQR)	245 (214,268)	222 (204,274)	376 (318,417)	273 (251,345)	448 (389,497)	374 (314,434)
Mean ± SD	245 ± 39	242 ± 56	364 ± 58	299 ± 57	455 ± 82	375 ± 62

Predicted PER = 32.5 + 197(BSA) – 32.4(gender); Root mean squared error (RMSE) = 67.5

Predicted PFR = 26.5 + 215(BSA) – 23.0(gender); RMSE = 79.6

Where gender is coded as 1 for male and 2 for female. The equations for Z-score calculation are:

Z_PER_ = (PER – (32.5 + 197(BSA) – 32.4(gender))) / 67.5

Z_PFR._ = (PFR – (26.5 + 215(BSA) – 23.0(gender))) / 79.6

Using the compressed method, PFR/EDV and PER/EDV were not significantly affected by BSA, gender, or age. PFR/EDV using the standard method was also not affected by BSA, gender, or age, but PER/EDV measured using the standard method demonstrated a small difference by gender (p = 0.02). Therefore, normative data for all of these metrics are stratified by gender (Table 7).

**Table 7:** Reference values for PFR and PER indexed to EDV, categorized by gender. IQR: interquartile range

	Male (n = 53)	Female (n = 43)
Standard method		
PFR/EDV		
Median (IQR)	3.2 (2.7,3.7)	3.6 (3.0,4.0)
Mean ± SD	3.2 ± 0.7	3.6 ± 0.7
PER/EDV		
Median (IQR)	3.2 (2.8,3.6)	3.3 (3.1,3.8)
Mean ± SD	3.2 ± 0.6	3.5 ± 0.5
Compressed method		
PFR/EDV		
Median (IQR)	3.1 (2.7,3.8)	3.4 (3.1,3.9)
Mean ± SD	3.2 ± 0.7	3.5 ± 0.7
PER/EDV		
Median (IQR)	3.0 (2.7,3.2)	3.0 (2.7,3.3)
Mean ± SD	3.0 ± 0.6	3.0 ± 0.5

Because tPER and tPFR were not significantly affected by BSA, gender or age, normal values are reported without further categorization (Table 8). LA volumes were not significantly affected by BSA, gender, or age (all p > 0.05). Thus, normative data for LA values are reported without categorization in Table 9.

**Table 8:** Reference values for tPFR and tPER. IQR: interquartile range

	tPER (ms)	tPFR (ms)
Standard method		
Median (IQR)	120 (101,140)	141 (125,162)
Mean ± SD	124 ± 32	145 ± 29
Compressed method		
Median (IQR)	127 (111,142)	125 (109,147)
Mean ± SD	128 ± 22	130 ± 28

**Table 9:** Reference values for LA volumes indexed to BSA and LA function

	LA_max_ (ml/m^2^)	LA_min_ (ml/m^2^)	LA_bac_ (ml/m^2^)	LAEF	LAEF_Passive_	LAEF_Active_
Median (IQR)	33 (29,38)	13 (11,16)	20 (18,26)	0.59 (0.54,0.65)	0.40 (0.28,0.46)	0.34 (0.27,0.43)
Mean ± SD	34 ± **7**	14 ± 4	22 ± 7	0.59 ± 0.09	0.36 ± 0.14	0.34 ± 0.11

### Correlations with other variables of interest

PFR correlated strongly with systolic blood pressure, but PFR/EDV did not (Additional file 1: Table S1). Indexed LA volumes only correlated weakly with systolic blood pressure. Multivariable linear regression demonstrated that PFR no longer correlated with systolic blood pressure once BSA was included in the model, suggesting that the blood pressure correlation with PFR was due to patient size. Similarly, PFR correlated with body mass index (BMI) but no longer had a significant association after correction for BSA. Heart rate did not correlate with PFR or tPFR, but correlated moderately with PFR/EDV. Further evaluation demonstrated a moderate correlation with LV end-diastolic volume (LVEDV) and indexed LVEDV (Rho = -0.47 and Rho = -0.49, p < 0.001 for both) suggesting that this correlation may be driven by LVEDV.

## Discussion

Ventricular filling curves and LA volumes calculated using CMR have significant potential for the non-invasive assessment of diastolic function in pediatric patients. However, these data have been limited by a scarcity of normative values and, in the case of LV filling curves, the time-intensive analysis process. Even with the use of artificial intelligence for automatic contouring of the ventricles, the time to complete diastolic filling curves likely prevents its routine use for clinical reports. This manuscript addresses these limitations by: (1) reporting normative data for LA function and ventricular filling curves in pediatric patients; (2) reporting an alternative, compressed method for ventricular filling curves that can be performed more quickly than the standard method. Importantly, the filling metrics derived from the compressed method correlate strongly with those from the standard method and are equally reproducible.

Several prior studies have reported normal values for biventricular EDV, end-systolic volume (ESV), stroke volume, wall mass and ejection fraction in children [16–19]. The most comprehensive review of normal CMR values by Kawel-Boehm et al. reports a summary of all available data for normal morphological and functional parameters in both children and adults [20]. The only prior evaluation of LA volumes in pediatric patients of which we are aware demonstrated relatively constant indexed LA volume in females, but a slight increase in the indexed volumes in males [12]. While we did not see a difference between genders in our data, there were methodological differences that may explain this discrepancy, including the inclusion of the appendage in their data and the use of a semi-automatic thresholding method for volumetric analysis as opposed to the biplane method. LA functional data have been well studied in adult populations. LAEF is of particular utility in evaluating diastolic dysfunction as it correlates with elevated LV end-diastolic pressure by invasive measures, and is an indicator of diastolic dysfunction [21, 22]. Similarly, in patients with hypertrophic cardiomyopathy, a decline in LA function is often observed prior to LA volume changes or myocardial hypertrophy [8]. Much of the current understanding of pediatric LA function is extrapolated from adult studies. This is the first study of which we are aware to report normative CMR LA functional data in pediatric patients.

There are no established pediatric reference ranges for LV filling curves of which we are aware. This is also the first report of our compressed method for calculating LV filling, which substantially decreases the analysis time for calculating LV filling. A recently published study by Kikano et al. highlights the association of CMR diastolic indices with arrhythmia in repaired tetralogy of Fallot. Prolonged tPFR and lower PFR/EDV were indicators of future arrhythmia development and associated with higher rates of mortality [9]. Abnormal CMR diastolic filling indices have also been previously associated with adverse outcomes in adults with hypertrophic cardiomyopathy, coronary artery disease, and varying degrees of heart failure [23–26]. In particular, prolonged tPFR and low PFR/EDV were shown to be highly sensitive and specific predictors of early LV diastolic dysfunction [24]. Prior adult data have demonstrated normal PFR in males and females that are comparable to our results in the largest BSA tertile from our study [27]. Interestingly, that same study reported nearly identical PFR/EDV, suggesting that this is relatively constant across age, BSA, and gender. Newly established normative pediatric data for LV filling and LA volumes and function may aid clinicians in more accurately evaluating diastolic function in a non-invasive manner, and potentially identifying early signs of diastolic dysfunction. This is of particular importance, as diastolic dysfunction is associated with adverse cardiac outcomes throughout the life span [2, 3, 5, 6]. Based on our findings, we expect that PFR and PFR/EDV will be the highest impact filling parameters, while LAEF will be the highest impact LA metric, in line with previous studies on diastolic function [9, 10, 21, 23, 25].

Both methods of diastology in this study have similar reproducibility, but our laboratory’s compressed method takes approximately half the time compared to standard methodology. Not surprisingly, the compressed method generates slower absolute filling and ejection rates when compared to the standard method. The most likely explanation for this difference is the relative differences in ventricular volumes that are used for rate calculation. The rate of ventricular filling is a product of ventricular volume and heart rate. Several slices are removed from the base and apex, resulting in a relatively smaller ventricular volume compared to the standard method. Furthermore, standard methodology will often add additional volume at the base during diastole, but not systole, due to the longitudinal motion of the heart. The combination of these two factors results in a disproportionally higher volume removed from the EDV volume measurements compared to ESV. Although there is a difference in the absolute values, it is important to note that the basic shapes of the LV filling curves are similar, which results in the strong ICC between the two methods. Thus, we propose that when used in a consistent manner, our compressed method remains an effective and highly reproducible approach to diastology that is less time intensive than standard methodology.

Prior work in adults with various heart diseases demonstrate an association between CMR derived values for PFR, tPFR, PFR/EDV and LAEF and elevated LV end-diastolic pressure [22, 28–30]. Data in pediatric heart transplant recipients also demonstrates an association between PFR and LA volumes with PCWP, though PCWP was measured under general anesthesia, which likely limited the association [8]. Given the complexity of diastole, future work should focus on understanding the relationship between CMR diastology metrics and those calculated from cardiac catheterization and echocardiography. A better understanding of factors affecting CMR diastology, such as BMI and heart rate, is also important. In addition, investigators should determine which heart diseases benefit the most from calculation of diastolic metrics.

## Limitations

This study has several limitations. Most notably, tPFR and tPER reproducibility is average to poor compared to the other indices that were highly reproducible. We hypothesize that the poor reproducibility is a consequence of the inherent limitations in CMR temporal resolution. A small difference in the slope of the curve could move the tPFR or tPER by 1–2 phases, thus resulting in a significant decrease in the correlation. Interpolation of phases may improve the reproducibility. Additionally, it was not possible in this retrospective study to obtain full atrial volumes with volumetry. Best estimations are reported with the area-length method, but there may be significant differences between these estimates and true volumes. Further investigation with a prospective cohort may improve LA volume analysis. Our study was also limited by sample size, though the total sample is similar to prior pediatric reports [17, 18, 31]. In particular, there was a small sample of children less than 10 years old and none below 7 years old, which is not an uncommon limitation in pediatric CMR studies. Therefore, use of reference values may not be as accurate in children younger than 7 years of age, and future studies should be undertaken to report normative data for this age group. Finally, given the relatively small sample size, Z-scores should be interpreted with caution.

## Conclusions

In conclusion, we report normative CMR LV filling data for pediatric patients as measured by two different methods. We also report normative data for LA volume and function. Our laboratory’s compressed method of generating LV filling curves is more rapid with similar results to the standard method and may facilitate the measurement of diastolic function in pediatric patients.

## Supplementary Information

**Additional file 1:** **Table S1.** Spearman Correlation, p-values, and N for CMR correlation with systolic blood pressure, heart rate, and body mass index. **Fig. S1.** Bland-Altman comparison of compressed and standard methods for calculation of **A** PFR, **B** tPFR, and **C** PFR/EDV. As expected, there is a significant bias for PFR and tPFR; the bias for PFR resolves with calculation of PFR/EDV. **Fig. S2.** Bland-Altman evaluation of inter-observer variability for **A** PFR, **B** tPFR, and **C** PFR/EDV for compressed method. **Fig. S3.** Bland-Altman evaluation of inter-observer variability for **A** PFR, **B** tPFR, and **C** PFR/EDV for standard method.

## Data Availability

The datasets generated and analyzed in this study are available from the corresponding author on reasonable request.

## Abbreviations

BMI: Body mass index
BSA: Body surface area
bSSFP: Balanced steady state free precession
CMR: Cardiovascular magnetic resonance
ECG: Electrocardiogram
EDV: End-diastolic volume
ESV: End-systolic volume
LV: Left ventricular
LA: Left atrium/left atrial
LAEF: Left atrial ejection fraction
LAEF_Active_: Left atrial ejection fraction during active phase
LAEF_Passive_: Left atrial ejection fraction during passive phase
LA_max_: Maximum left atrial volume
LA_min_: Minimum left atrial volume
LA_bac_: Pre-atrial contraction left atrial volume
LV: Left ventricle/left ventricular
LVEDV: Left ventricular end-diastolic volume
PCWP: Pulmonary capillary wedge pressure
PER: Peak ejection rate
PFR: Peak filling rate
tPER: Time to peak ejection rate
tPFR: Time to peak filling rate

## References

[1] Singh GK, Holland MR (2010). Diastolic dysfunction in pediatric cardiac patients: evaluation and management. Curr Treat Options Cardio Med.

[2] McMahon CJ, Nagueh SF, Pignatelli RH, Denfield SW, Dreyer WJ, Price JF (2004). Characterization of left ventricular diastolic function by tissue Doppler imaging and clinical status in children with hypertrophic cardiomyopathy. Circulation.

[3] Norrish G, Cantarutti N, Pissaridou E, Ridout DA, Limongelli G, Elliott PM (2017). Risk factors for sudden cardiac death in childhood hypertrophic cardiomyopathy: a systematic review and meta-analysis. Eur J Prev Cardiolog.

[4] Banarjee. Effect of myocardial hypertrophy on systolic and diastolic function in children: insights from the force-frequency and relaxation-frequency relationships—ScienceDirect [Internet]. https://www.sciencedirect.com/science/article/pii/S0735109798003507. Accessed 3 Feb 2022. 10.1016/s0735-1097(98)00350-7 9768737

[5] Earing MG, Cetta F, Driscoll DJ, Mair DD, Hodge DO, Dearani JA (2005). Long-term results of the Fontan operation for double-inlet left ventricle. The Am J Cardiol.

[6] McMahon CJ, Pignatelli RH, Nagueh SF, Lee VV, Vaughn W, Valdes SO (2007). Left ventricular non-compaction cardiomyopathy in children: characterisation of clinical status using tissue Doppler-derived indices of left ventricular diastolic relaxation. Heart.

[7] Mawad W, Friedberg MK (2017). The continuing challenge of evaluating diastolic function by echocardiography in children: developing concepts and newer modalities. Curr Opin Cardiol.

[8] Belay W, Godown J, Chan KC, Bearl DW, George-Durrett K, Slaughter JC (2022). Cardiac magnetic resonance diastolic indices correlate with ventricular filling pressures in pediatric heart transplant recipients. Pediatr Transplant.

[9] Kikano S, Weingarten A, Sunthankar SD, McEachern W, George-Durett K, Parra DA (2023). Association of CMR diastolic indices with arrhythmia in repaired tetralogy of Fallot. J Cardiovascr Magn Reson..

[10] Farhad H, Seidelmann SB, Vigneault D, Abbasi SA, Yang E, Day SM (2017). Left Atrial structure and function in hypertrophic cardiomyopathy sarcomere mutation carriers with and without left ventricular hypertrophy. J Cardiovasc Magn Reson..

[11] Dodson JA, Neilan TG, Shah RV, Farhad H, Blankstein R, Steigner M (2014). Left atrial passive emptying function determined by cardiac magnetic resonance predicts atrial fibrillation recurrence after pulmonary vein isolation. Circ Cardiovasc Imaging.

[12] Sarikouch S, Koerperich H, Boethig D, Peters B, Lotz J, Gutberlet M (2011). Reference values for atrial size and function in children and young adults by cardiac MR: a study of the German competence network congenital heart defects. J Magn Reson Imaging.

[13] Rodríguez-Granillo GA, Mejía-Campillo M, Rosales MA, Bolzán G, Ingino C, López F (2012). Left ventricular filling patterns in patients with previous myocardial infarction measured by conventional cine cardiac magnetic resonance. Int J Cardiovasc Imaging.

[14] Chacko BR, Karur GR, Connelly KA, Yan RT, Kirpalani A, Wald R (2018). Left ventricular structure and diastolic function by cardiac magnetic resonance imaging in hypertrophic cardiomyopathy. Indian Heart J.

[15] The Correlation Coefficient (r) [Internet]. https://sphweb.bumc.bu.edu/otlt/MPH-Modules/PH717-QuantCore/PH717-Module9-Correlation-Regression/PH717-Module9-Correlation-Regression4.html. Accessed 21 Feb 2023.

[16] van der Ven JPG, Sadighy Z, ValsangiacomoBuechel ER, Sarikouch S, Robbers-Visser D, Kellenberger CJ (2020). Multicentre reference values for cardiac magnetic resonance imaging derived ventricular size and function for children aged 0–18 years. Euro Heart J - Cardiovasc Imaging.

[17] Olivieri LJ, Jiang J, Hamann K, Loke YH, Campbell-Washburn A, Xue H (2020). Normal right and left ventricular volumes prospectively obtained from cardiovascular magnetic resonance in awake, healthy, 0–12 year old children. J Cardiovasc Magn Reson.

[18] Sarikouch S, Peters B, Gutberlet M, Leismann; B, Kelter-Kloepping A, Koerperich H, et al. Sex-specific pediatric percentiles for ventricular size and mass as reference values for cardiac MRI: assessment by steady-state free-precession and phase-contrast MRI flow. Circ Cardiovasc Imaging. 2010;3(1):65–76. 10.1161/CIRCIMAGING.109.859074 19820203

[19] Helbing WA, Rebergen SA, Maliepaard C, Hansen B, Ottenkamp J, Reiber JHC (1995). Quantification of right ventricular function with magnetic resonance imaging in children with normal hearts and with congenital heart disease. Am Heart J.

[20] Kawel-Boehm N, Hetzel SJ, Ambale-Venkatesh B, Captur G, Francois CJ, Jerosch-Herold M (2020). Reference ranges (“normal values”) for cardiovascular magnetic resonance (CMR) in adults and children: 2020 update. J Cardiovasc Magn Reson.

[21] Aquaro GD, Pizzino F, Terrizzi A, Carerj S, Khandheria BK, Di Bella G (2019). Diastolic dysfunction evaluated by cardiac magnetic resonance: the value of the combined assessment of atrial and ventricular function. Eur Radiol.

[22] Posina K, McLaughlin J, Rhee P, Li L, Cheng J, Schapiro W (2013). Relationship of phasic left atrial volume and emptying function to left ventricular filling pressure: a cardiovascular magnetic resonance study. J Cardiovasc Magn Reson.

[23] Kawaji K, Codella NCF, Prince MR, Chu CW, Shakoor A, LaBounty TM (2009). Automated segmentation of routine clinical cardiac magnetic resonance imaging for assessment of left ventricular diastolic dysfunction. Circ Cardiovasc Imaging.

[24] Xu H, Yang Z, Guo Y, Shi K, Liu X, Zhang Q (2017). Volume-time curve of cardiac magnetic resonance assessed left ventricular dysfunction in coronary artery disease patients with type 2 diabetes mellitus. BMC Cardiovasc Disorders..

[25] Chen X, Hu H, Qian Y, Shu J (2014). Relation of late gadolinium enhancement in cardiac magnetic resonance on the diastolic volume recovery of left ventricle with hypertrophic cardiomyopathy. J Thorac Dis.

[26] Hieda M, Parker J, Rajabi T, Fujimoto N, Bhella PS, Prasad A (2018). Left ventricular volume-time relation in patients with heart failure with preserved ejection fraction. Am J Cardiol.

[27] Maceira AM, Prasad SK, Khan M, Pennell DJ (2006). Reference right ventricular systolic and diastolic function normalized to age, gender and body surface area from steady-state free precession cardiovascular magnetic resonance. Eur Heart J.

[28] Patel D, Robinson VJB, Arteaga RB, Thornton JW (2008). Diastolic filling parameters derived from myocardial perfusion imaging can predict left ventricular end-diastolic pressure at subsequent cardiac catheterization. J Nucl Med.

[29] Rokey R, Kuo LC, Zoghbi WA, Limacher MC, Quinones MA (1985). Determination of parameters of left ventricular diastolic filling with pulsed Doppler echocardiography: comparison with cineangiography. Circulation.

[30] Zile MR, Brutsaert DL (2002). New concepts in diastolic dysfunction and diastolic heart failure: Part I: diagnosis, prognosis, and measurements of diastolic function. Circulation.

[31] van der Ven JPG, Sadighy Z, ValsangiacomoBuechel ER, Sarikouch S, Robbers-Visser D, Kellenberger CJ (2020). Multicentre reference values for cardiac magnetic resonance imaging derived ventricular size and function for children aged 0–18 years. Eur Heart J - Cardiovasc Img.

