# Supplementary material for: Reference data for left ventricular filling and atrial function in children using cardiovascular magnetic resonance
Source: J Cardiovasc Magn Reson. 2023 Jun 12;25:30. doi: 10.1186/s12968-023-00936-x (PMC10258747; doi:10.1186/s12968-023-00936-x)

**Additional file 1: Table S1:** Spearman Correlation, p-values, and N for CMR correlation with systolic blood pressure, heart rate (at time of short axis stack), and body mass index

|  | **Systolic blood pressure** | **Heart rate** | **Body mass index** |
| --- | --- | --- | --- |
| **Compressed**  **Method** |  |  |  |
| **PFR** | Rho=0.45  P<0.001  N=64 | Rho=-0.13  P=0.19  N=96 | Rho=0.61  P<0.001  N=96 |
| **tPFR** | Rho=-0.02  P=0.86  N=64 | Rho=-0.27  P=0.007  N=96 | Rho=-0.05  P=0.66  N=96 |
| **PFR/EDV** | Rho=-0.10  P=0.42  N=64 | Rho=0.47  P<0.001  N=96 | Rho=0.03  P=0.78  N=96 |
| **Standard**  **Method** |  |  |  |
| **PFR** | Rho=-0.36  P=0.004  N=64 | Rho=-0.16  P=0.12  N=96 | Rho=0.62  P<0.001  N=96 |
| **tPFR** | Rho=-0.14  P=0.26  N=64 | Rho=-0.12  P=0.24  N=96 | Rho=-0.12  P=0.26  N=96 |
| **PFR/EDV** | Rho=-0.20  P=0.11  N=64 | Rho=0.49  P<0.001  N=96 | Rho=-0.03  P=0.79  N=96 |
| **LA_max_ volume indexed** | Rho=0.21  P=0.09  N=64 | Rho=-0.22  P=0.03  N=96 | Rho=0.09  P=0.40  N=96 |
| **LA_min_ volume indexed** | Rho=0.29  P=0.03  N=64 | Rho=-0.41  P<0.001  N=96 | Rho=0.14  P=0.17  N=96 |
| **LA_bac_ volume indexed** | Rho=0.29  P=0.02  N=64 | Rho=-0.20  P=0.05  N=96 | Rho=0.15  P=0.14  N=96 |

**Additional file 1: Fig. S1:** Bland-Altman comparison of compressed and standard methods for calculation of A) PFR, B) tPFR, and C) PFR/EDV. As expected, there is a significant bias for PFR and tPFR; the bias for PFR resolves with calculation of PFR/EDV.


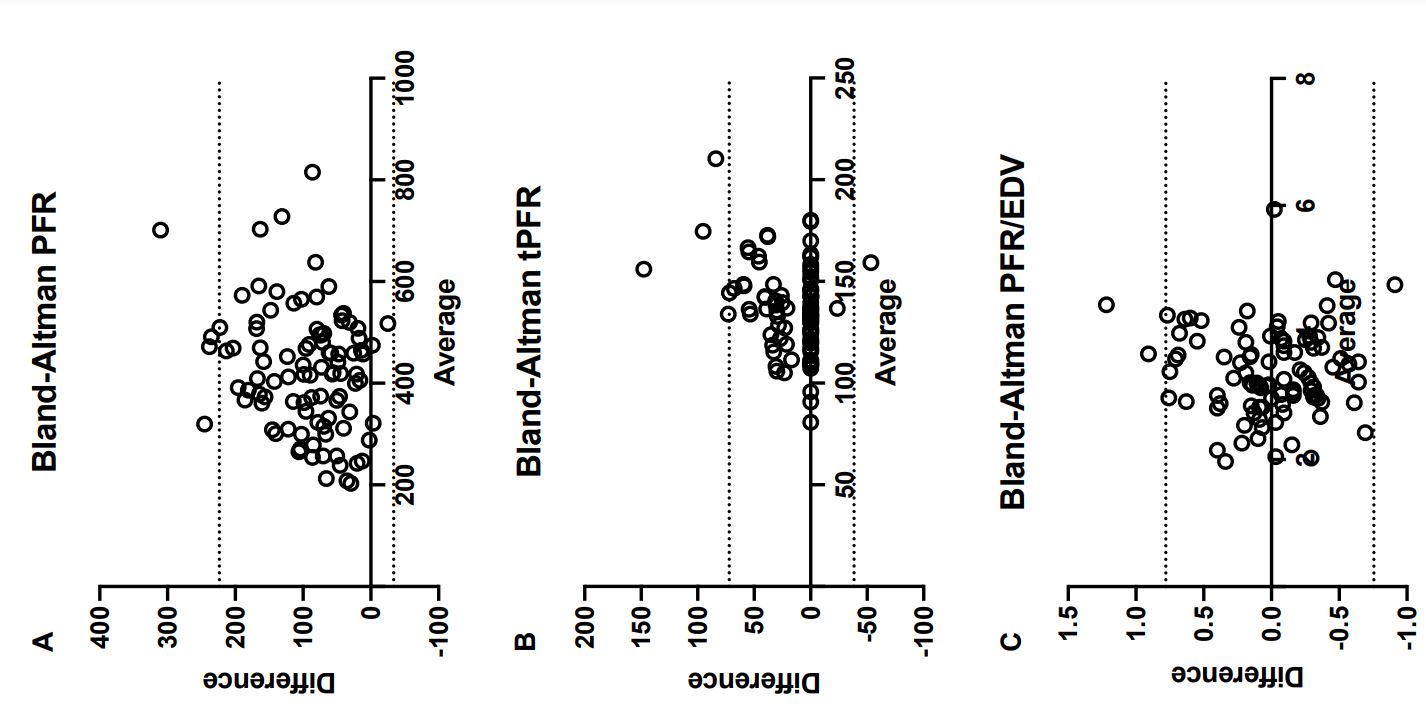


**Additional file 1: Fig. S2**: Bland-Altman evaluation of inter-observer variability for A) PFR, B) tPFR, and C) PFR/EDV for compressed method.


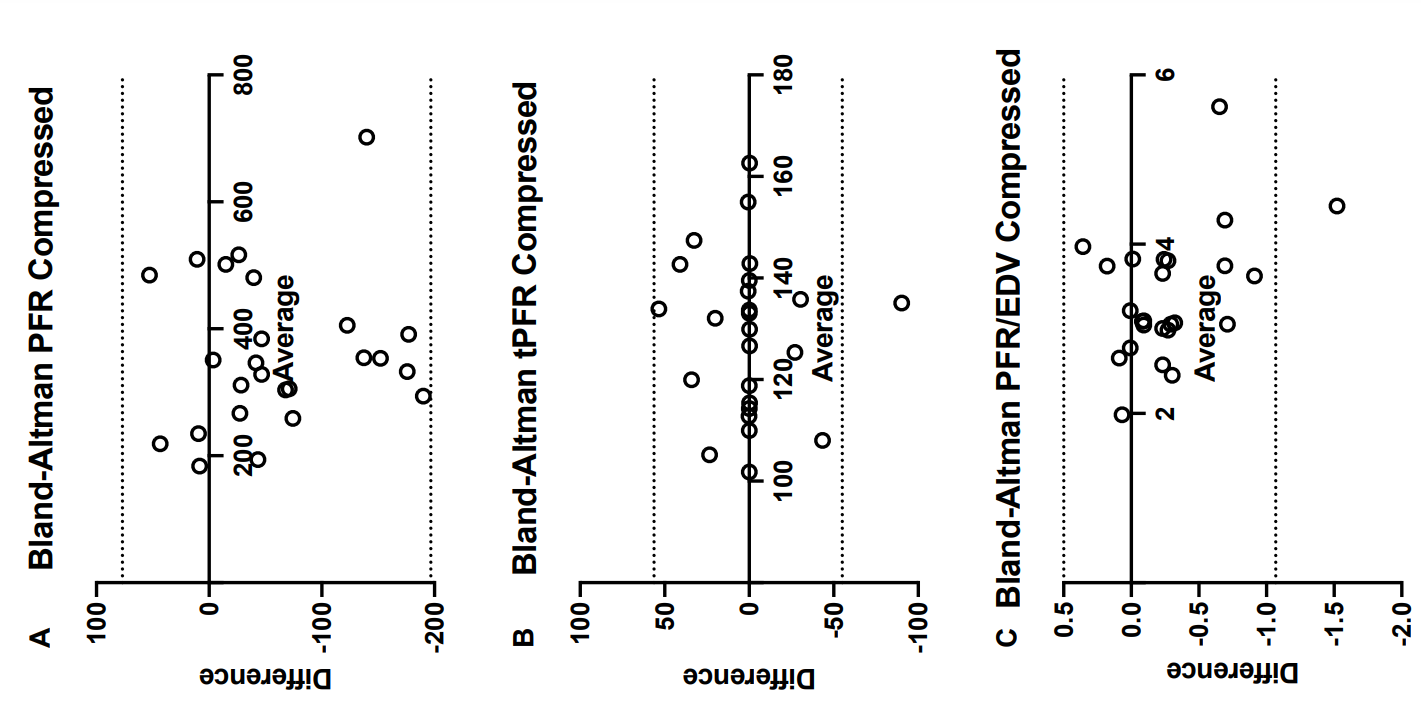


**Additional file 1: Fig. S3:** Bland-Altman evaluation of inter-observer variability for A) PFR, B) tPFR, and C) PFR/EDV for standard method.


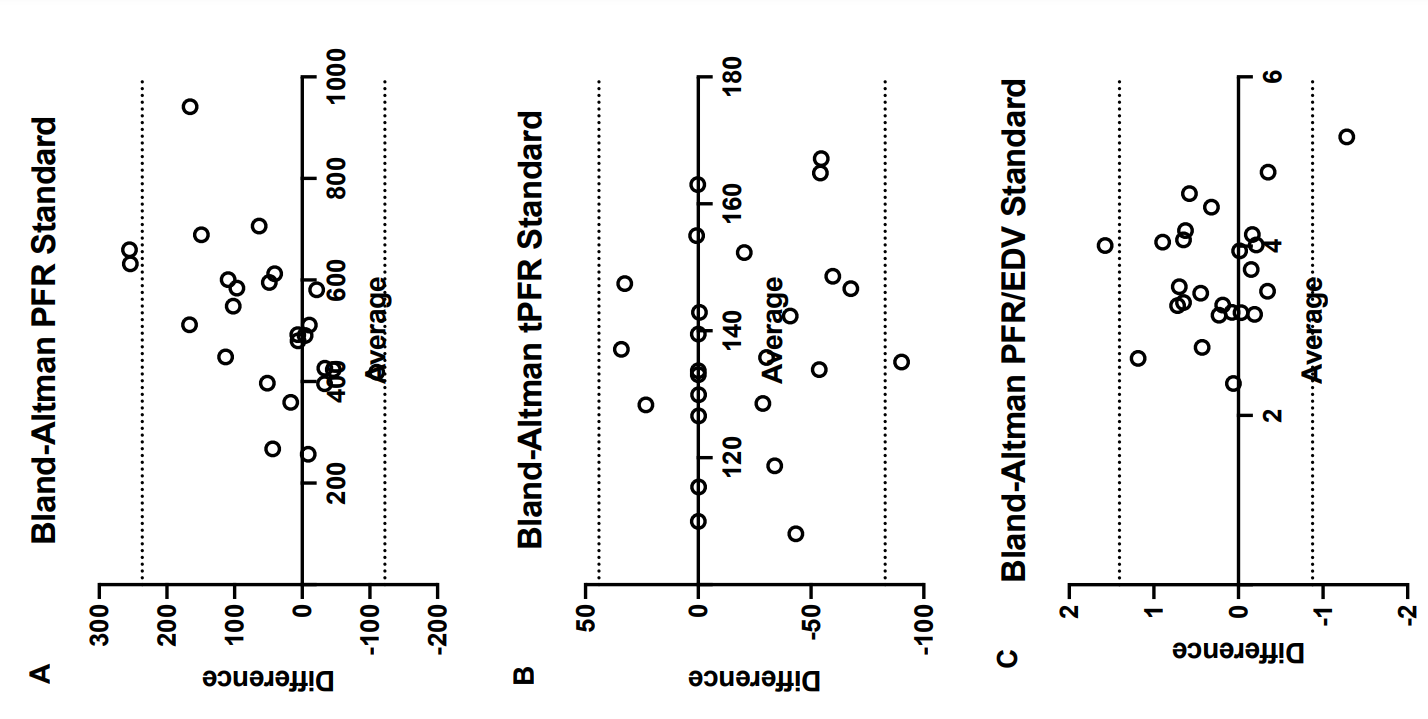

Supplement: Supplementary file 1 — Additional file 1: Table S1. Spearman Correlation, p-values, and N for CMR correlation with systolic blood pressure, heart rate, and body mass index. Fig. S1. Bland-Altman comparison of compressed and standard methods for calculation of A PFR, B tPFR, and C PFR/EDV. As expected, there is a significant bias for PFR and tPFR; the bias for PFR resolves with calculation of PFR/EDV. Fig. S2. Bland-Altman evaluation of inter-observer variability for A PFR, B tPFR, and C PFR/EDV for compressed method. Fig. S3. Bland-Altman evaluation of inter-observer variability for A PFR, B tPFR, and C PFR/EDV for standard method. [file 12968_2023_936_MOESM1_ESM.docx]
